# Genetic composition of Kazakh horses of Zhabe type evaluated by SNP genotyping

**DOI:** 10.1016/j.heliyon.2024.e41173

**Published:** 2024-12-12

**Authors:** Alexandr Pozharskiy, Indira Beishova, Askar Nametov, Alzhan Shamshidin, Tatyana Ulyanova, Alexandr Kovalchuk, Vadim Ulyanov, Malika Shamekova, Gulmira Bekova, Dilyara Gritsenko

**Affiliations:** a Institute of Plant Biology and Biotechnology, Timiryazev Str. 45, 050040, Almaty, Kazakhstan; b Al-Farabi Kazakh National University, Al-Farabi Ave. 71, 050040, Almaty, Kazakhstan

**Keywords:** *Equus caballus*, GWAS, Body weight, Body size, Selection signatures

## Abstract

Horses are animals traditionally playing prominent role as both food source and working animals for Kazakh people. Zhabe horses are traditional type of indigenous Kazakh horses characterized by versatility and adaptation to conditions of Central Asia. The present work focuses on examination of genetic structure of Zhabe horses using SNP genotyping with addition of previously published data. Total 1038 individuals including 403 new samples of Zhabe horses and 42 sample of white horses ‘Zhetysu Asyly’ have been considered. DNA was extracted from hair roots using commercial DNA isolation kit and further used for analysis of SNP by Illumina iScan system with Equine80k SNP array. The analysis of population genetic parameters (expected and observed heterozygosity, linkage disequilibrium, Wright's *F*_*st*_) and genetic structure (PCA, ADMIXTURE) in comparison with publicly available data on selected foreign cultivars demonstrated low between population differentiations and lack of selection factors. Genome wide association study performed for body size and weight have revealed low occurrence of SNPs with significant associations, total 57 SNPs linked to various genes with low density across all genome. The obtained results highlight difference between traditional horse breeding practices of Kazakh people and stable based breeding of foreign breeds. In contrast, the ‘Zhetysu Asyly’ horse breed derived from Kazakh horses demonstrate the effect of intense breeding process on the same landrace. The results provide new data on the traditional Kazakh horses of type Zhabe and will assist further studies of this original landrace.

## 1 Introduction

Since the ancient time, domestic horses *Equus caballus* L. have been an essential part of economics of Kazakhstan as both a source of food and saddle/working animals. The area of Kazakhstan is arguably one of the place where horse domestication could take place historically, as the most ancient traces of horse have been discovered in this region [[1], [2], [3], [4]]. The environment of the Central Asian steppes and the traditional ways of horse husbandry of local nomadic peoples have formed a specific type of domestic horse, namely Kazakh horse. The traditional Kazakh horses include three main types Zhabe, Adai, and Naiman, which originated from different regions of the country. Based on them, more derivative breeds were developed; the most known of them include Kostanay, Kushum, Mugalzhar and other breeds [[5], [6], [7]].

The most known horses of the Kazakh breed are Zhabe horses. Zhabe horses have originated from southern parts of Aktobe region and distributed all over Kazakhstan. Their origin remains disputable; main hypotheses include the origination from wild Asian horses or Mongolian horse breed, or that they gradually evolved under combination of multiple factors of the natural and artificial selection [8]. Supposedly, Kazakh horses have been influenced by Mongolian, Karabair, Arabian, and Akhal-Teke horse breeds; the modern influence (20th century) include also the Thoroughbred, Orlov Trotter and Don horses [6]. Zhabe horses are considered to be a typical Kazakh horse landrase as they are the most widespread and not specialized for some specific conditions and/ar application (comparing to horses of Naiman type better adopted to mountain regions [7], or Adai horses which have been specialized as saddle horse [6]).

Zhabe are characterized by rugged head, thick neck, wide body with straight back, deep chest, and muscled croup; skin is dense; hair colors varying from light gray to dark bay or red. Zhabe horses have relatively high body weight (400–500 kg) for their size (height at withers 142–144 cm, chest circumference 178–180 cm, cannon bone circumference 18.8–19 cm [6]. Young Zhabe horses intensively accumulate living weight during pasturing and produce high quality meat regardless of their population and regional lineage [9]. Their body properties and good milk productivity make Zhabe horses an important resource for meat and milk production. Zhabe horses are well adapted to the traditional nomadic husbandry practices based on seasonal transhumance and pasturing [8]; such way of horse breeding have been developing since Saka-Skythian historic period (mid-first millennium BC) [10].

The traditional methods of horse breeding have been based on simple selection and crossing of animals with desirable traits. Although such methods are still in use in horse selection programs, their sole usage limits the breeding progress. Modern practices imply the use of the data on genetic mechanisms underlying economically import traits such as productivity. Genomic and marker associated selection (MAS) became an important field of animal breeding as the increasing data on genetic polymorphism stimulate studies not only elucidating genetic properties of breeds but also endorsing them for use in selection practice. The study of breeds, using molecular techniques is very important and useful for their characterizing [[11], [12], [13]]. Conservation of genetic diversity in animal species requires the proper performance of conservation superiorities and sustainable handling plans that should be based on universal information on population structures, including genetic diversity resources among and between breeds [14,15]. Genetic diversity is an essential element for genetic improvement, preserving populations, evolution and adapting to variable environmental situations [16,17]. On the other hands, determination of gene polymorphism is important in farm animals breeding [18,19] in order to define genotypes of animals and their associations with productive, reproductive and economic traits [[20], [21], [22]].

Considering meat productivity, the living mass and size of an animal are the primary traits of interest. Body mass is a multifactorial quantitative trait affected by both genetic and ambiance factors. For example, heritability of weight and body measures have been shown previously [[23], [24], [25]]. Particularly, heritability of Zhabe body mass and measurements have been estimated [26,27]. The more detailed studied on genetic factors affecting horse body parameters involve the advances of horse genomics. The availability of continuously updated reference assembly of horse genome (current version EquCab3.0 [28]) assists studies on genetic bases of various traits of interest involving thousands and millions of SNPs [29,30]. Genome wide association studies (GWAS) have identified genes LCORL/NCAPG, HMGA2, ZFAT, and LASP1 as the major genetic factors associated with body size in horses [[31], [32], [33], [34]].

To date, despite their prominent economical role in the country, only limited number of studies on the molecular genetic background of Kazakh horses including Zhabe have been conducted. The most used molecular tool is microsatellite markers, or short tandem repeats (STR). For example, STR-markers were used to investigate genetic structure of Adai horses [35] and Mugalzhar horse breed [36].

Our previous study was the biggest to date and involved total 2020 horses of six traditional types and breeds of Kazakh horses analyzed using 80,000 SNP microarray [37]. This study have revealed the surprising absence of genetic differentiation between studied breeds. The observed genetic structure was an evidence for existence of broadly defined landrace corresponding to Kazakh horses without notable genetic demarcation between rational types and breeds. It has been also hypothesized that the traditional nomadic ways of horse breeding on the territory of contemporary Kazakhstan could have lead to formation of Kazakh horse landrace without strong pressure of artificial selection [37]. The present article is focused on horses of Zhabe type and uses previously published data combined with newly genotyped sample of Zhabe horses to provide more details on the genetic structure is type of Kazakh horses including GWAS for body weight and size, the traits associated with meat productivity. A special attention was paid to population genetic parameters potentially reflecting the way of horse selection.

## 2 Materials and methods

### 2.1 Sample collection and DNA isolation

Genetic material (hairs from tails and/or manes) of Zhabe horses was collected at private farms “Alakol Asyl” (Zhetysu region) and “Akzhar” (Pavlodar region); additionally, samples of white horses “Zhetysu Asyly” were collected at A. Sophin's private farm (Zhetysu region) (Table 1). All collected materials were stored at 4 °C until further use. DNA was isolated from hair follicles using the kit ‘‘DNK-Extran2” (Syntol, Russian Federation) following the manufacturer's protocol and quantified using Qubit 4 Fluoremeter with the Qubit dsDNA Broad Range reagent (Thermo Fisher Scientific, USA) for downstream analysis.

**Table 1:** Summary of samples and data used in the study.

Zhabe horses, Kazakhstan						
Population code	Husbandry	Region	Number of individuals with available data		Final number of individuals used for analysis	Data source
			Genotype	Phenotype (weight, size)		
37	Alakol Asyl	Zhetysu^a^	421	427	384	Present study
39^b^	A. Sophin	Zhetysu^a^	42	88	42	
25	Akzhar	Pavlodar	81^c^	173	64	[37], present study
33	Agro-Damu	Pavlodar	153	186	152	[37]
3	Kalka	Zhambyl	143	163	141	
28	Zhaksylyk	Almaty	86	122	86	
34	Anar	Pavlodar	76	77	76	
32	Sayakhat	Almaty	48	0	47	
29	Kyzylsok	Almaty	46	46	46	
		Total	1096	1282	1038	
Foreign breeds						
Breed code	Breed name	Breed origin	Number of genotypes		Sample origin	Data source

AKTK	Akhat-Teke	Turkmenistan	19		USA, Russia	[41]
ARR	Arabian	Middle East	24		USA	
CSP	Caspian	Persia	19		USA	
MON	Mongolian	Mongolia	19		Mongolia	
TB_UK	Thoroughbred	UK	19		UK	
TB_US			17		USA	

a Former part of Almaty region.

b Horses of the derivative “Zhetysu Asyly” breed.

c Including 17 newly analyzed samples.

Phenotypic data including body weight, height at the withers (HW), oblique body length (OBL), chest circumference (CC), and cannon bone circumference (CBC) were taken and provided by the horses owners prior to sample collection.

### 2.2 SNP genotyping

SNP genotyping of collected samples was performed using Equine80k SNP array on the iScan system (Illumina, USA) according to the manufacturer's protocol. Genotype calling and primary data filtering were performed using GenomeStudio software (Illumina, USA). The primary filtering criteria included call rate ≥0.9, median GC score ≥0.8 for samples; call frequency ≥0.95 and GT score ≥0.7 for SNP; indel markers included into array were excluded. PLINK1.9 software [38] was used for further data filtering to exclude SNPs with minor allele frequencies <0.05 and those deviating from Hardy-Weinberg equilibrium with a P-value threshold of 1 × 10^−5^. Additional sample filtering was performed based on data availability for particular individuals when needed.

### 2.3 Data analysis

Newly obtained genotyping data were combined with previously reported data on Zhabe horses [37]. Previously obtained unfiltered data were merged with current results and filtered (SNP call rate ≥0.9, minor allele frequency ≥0.05, Hardy-Weinberg equilibrium under *p-*value threshold 10^−5^). The obtained dataset was used for genetic structure analysis using ADMIXTURE software [39] and GWAS using PLINK1.9. ADMIXTURE run was set for *K* numbers of clusters from 2 to 20 with ten-fold cross-validation error estimate; results were vizualized using CLUMPAK web server [40].

For comparative analyzis the dataset on Zhabe horses was further merged with data on foreign horse breeds [41] and filtered anew with the same criteria. The following breeds were chosen: Akhal-Teke, Caspian, Arabian, Mongolian, and Thorougbred (two populations)(Table 1). The analysis included principal component analysis and evaluation of linkage disequilibrium (LD), expected and observed heterozygosity, and pairwise between population Wright's *F*_*ST*_ using PLINK1.9 and R [42]. Neighbor net for pairwise *F*_*ST*_ between Zhabe populations and foreign breeds was calculated and plotted using R package phangorn [43]. Additionally, ADMIXTURE analysis was performed for the combined dataset with the same running conditions.

Genome wide association study was conducted using general linear model implemented in PLINK1.9. Sex and age of animals in years were used as covariates of the model. Animals without available phenotypic data (e.g. population 32 “Sayakhat”) or identified as outlying genotypes by population structure analysis were excluded from the analysis. The four size measurements (HW, OBL, CC, and CBC) were normalized by subtracting the mean and dividing by standard deviation and then used to perform principal component analysis using built-in R functions. The first principal component was further used in GWAS as a synthetic variable describing animal body size. GWAS was performed separately for animal body weight and size using --linear command of PLINK with age and sex as covariates (model *Y* = *b*_*0*_ *+ b*_*1*_*X*_*ADD*_ *+* *b*_*2*_*X*_*AGE*_ *+ b*_*3*_*X*_*SEX*_ *+* *e,* where *Y –* phenotype*, X*_*ADD*_ – genotypes encoded as additive model (0, 2 for homozygote, 1 for heterozygote), *X*_*AGE*_ – covariate of age, *X*_*SEX*_ – covariate for sex, *b*_*n*_ – regression coefficients, *e* – residual error term). *P-*values were estimated using 1000 Monte-Carlo permutation test with adaptive number of permutations (PLINK's--perm command) and used to construct Manhattan plot using ‘ggplot2’ R package. Variant Effect Predictor [44] and DAVID [45,46] tools were used to annotate significant SNPs. Results for SNPs associated with body weight and size were combined and filtered to include only variants with available annotations and exclude variants with duplicate annotations.

## 3 Results

As a final result of genotyping and data filtering, genotypes of 443 horses have been obtained including 401 samples of Zhabe horses and 42 samples of white horses ‘Zhetysu Asyly’, in addition to previously obtained data [37] (Table 1). The final combined dataset included 1038 individuals with 43,422 SNPs. The combined dataset including five foreign breeds consisted of 24,764 SNP after additional filtering.

**Fig. 1:**
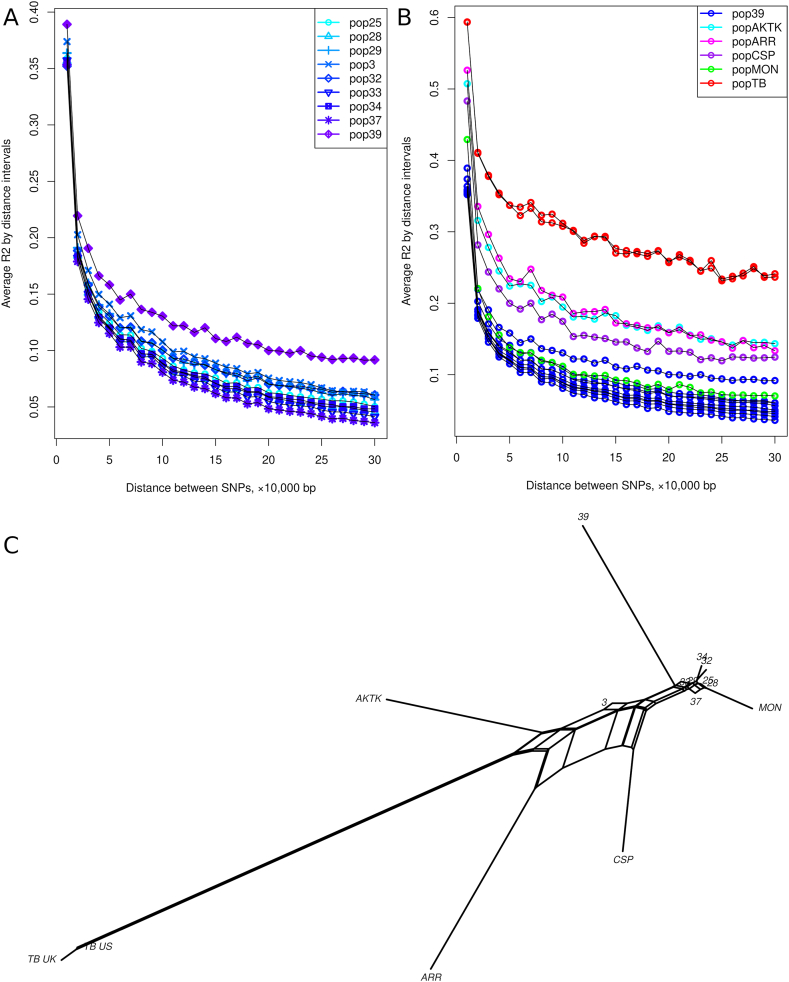
Plot of linkage disequilibrium and pairwise *F*_*st*_ of Zhabe horses; A) Linkage disequilibrium decay in nine studied populations of Zhabe horses; B) Linkage disequilibrium decay in Zhabe horses in comparison with Akhal-Teke, Caspian, Mongol, Arabian, and Thoroughbred horses; C) Neighbor net plot of pairwise *FST* between Zhabe horse populations and foreign breeds.

Calculated values of expected/observed heterozygosity and pairwise between population *F*_*ST*_ are shown in Table 2. Among Kazakh horses, the lowest hetezygosity level was in population 39 containing Zhetysu Asyly horses; expected value per chromosome 0.28 (SD 0.010), observed value 0.308 (SD 0.12). Other populations corresponding attributed Zhabe horses displayed expected values from 0.314 in population 34 to 0.334 in population 3, and observed values from 0.317 in populations 33 and 34 to 0.342 in population 32; the former population had the biggest deviation between expected and observed heterozygosity whereas other populations had only slightly differing values. All Kazakh horse populations had low *F*_*ST*_ values not exceeding 0.010 between each other except for population 39 with *F*_*ST*_ from 0.027 to 0.034. The foreign breeds displayed higher coefficients when compared to Zhabe horses: from 0.006 to 0.030 for Mongolian horses to 0.095–0.106 for the Thoroughbred horses. Thus, the Mongolian horses displayed more similarity to Zhabe horses, and the Thoroughbred horses were the most dissimilar. As it can be seen in the neighbor net plot of *F*_*ST*_ (Fig. 1, C), populations of Zhabe horses form close group except for popultaion 3 which demonstrated shift towards Thoroughbred horses. Population 39 (Zhetysu Asyly) formed independent branch distant from Zhabe populations.

**Table 2:** Population genetic parameters calculated for combined dataset (24,764 SNP).

Population	*N*	*H*_*Echr*_	*H*_*Ochr*_	*F*_*ST*_														
				25	28	29	32	33	34	37	39	3	AKTK	ARR	CSP	MON	TB_UK	TB_US
25	64	0.319 ± 0.009	0.325 ± 0.009	–	0.001 ± 0.001	0.003 ± 0.001	0.003 ± 0.001	0.003 ± 0.001	0.003 ± 0.001	0.003 ± 0.001	0.030 ± 0.007	0.008 ± 0.002	0.044 ± 0.006	0.057 ± 0.006	0.029 ± 0.004	0.008 ± 0.003	0.099 ± 0.017	0.096 ± 0.017
28	86	0.318 ± 0.008	0.318 ± 0.010	0.001 ± 0.001	–	0.004 ± 0.001	0.005 ± 0.001	0.002 ± 0.001	0.002 ± 0.001	0.003 ± 0.001	0.030 ± 0.007	0.008 ± 0.002	0.045 ± 0.007	0.058 ± 0.007	0.029 ± 0.004	0.007 ± 0.002	0.101 ± 0.018	0.098 ± 0.017
29	46	0.318 ± 0.009	0.326 ± 0.009	0.003 ± 0.001	0.004 ± 0.001	–	0.004 ± 0.001	0.003 ± 0.001	0.006 ± 0.001	0.004 ± 0.001	0.030 ± 0.007	0.007 ± 0.001	0.044 ± 0.006	0.057 ± 0.007	0.029 ± 0.005	0.008 ± 0.003	0.095 ± 0.015	0.092 ± 0.016
32	47	0.320 ± 0.009	0.342 ± 0.011	0.003 ± 0.001	0.005 ± 0.001	0.004 ± 0.001	–	0.006 ± 0.001	0.004 ± 0.001	0.006 ± 0.001	0.034 ± 0.007	0.011 ± 0.001	0.047 ± 0.006	0.059 ± 0.006	0.032 ± 0.004	0.012 ± 0.002	0.098 ± 0.017	0.095 ± 0.018
33	152	0.321 ± 0.009	0.317 ± 0.010	0.003 ± 0.001	0.002 ± 0.001	0.003 ± 0.001	0.006 ± 0.001	–	0.002 ± 0.001	0.002 ± 0.000	0.028 ± 0.007	0.006 ± 0.001	0.042 ± 0.006	0.055 ± 0.006	0.026 ± 0.004	0.006 ± 0.002	0.097 ± 0.016	0.094 ± 0.016
34	76	0.314 ± 0.009	0.317 ± 0.009	0.003 ± 0.001	0.002 ± 0.001	0.006 ± 0.001	0.004 ± 0.001	0.002 ± 0.001	–	0.004 ± 0.001	0.030 ± 0.006	0.010 ± 0.002	0.048 ± 0.006	0.061 ± 0.007	0.031 ± 0.005	0.007 ± 0.002	0.106 ± 0.018	0.104 ± 0.017
37	384	0.321 ± 0.008	0.319 ± 0.009	0.003 ± 0.001	0.003 ± 0.001	0.004 ± 0.001	0.006 ± 0.001	0.002 ± 0.000	0.004 ± 0.001	–	0.027 ± 0.007	0.008 ± 0.002	0.043 ± 0.006	0.057 ± 0.007	0.027 ± 0.004	0.005 ± 0.002	0.101 ± 0.017	0.099 ± 0.017
39	42	0.298 ± 0.010	0.308 ± 0.012	0.030 ± 0.007	0.030 ± 0.007	0.030 ± 0.007	0.034 ± 0.007	0.028 ± 0.007	0.030 ± 0.006	0.027 ± 0.007	–	0.032 ± 0.006	0.069 ± 0.009	0.079 ± 0.011	0.054 ± 0.007	0.034 ± 0.007	0.122 ± 0.017	0.120 ± 0.018
3	141	0.334 ± 0.008	0.324 ± 0.008	0.008 ± 0.002	0.008 ± 0.002	0.007 ± 0.001	0.011 ± 0.001	0.006 ± 0.001	0.010 ± 0.002	0.008 ± 0.002	0.032 ± 0.006	–	0.037 ± 0.006	0.050 ± 0.006	0.028 ± 0.004	0.015 ± 0.003	0.064 ± 0.012	0.060 ± 0.011
AKTK	19	0.300 ± 0.009	0.299 ± 0.012	0.044 ± 0.006	0.045 ± 0.007	0.044 ± 0.006	0.047 ± 0.006	0.042 ± 0.006	0.048 ± 0.006	0.043 ± 0.006	0.069 ± 0.009	0.037 ± 0.006	–	0.067 ± 0.009	0.058 ± 0.008	0.056 ± 0.009	0.097 ± 0.016	0.094 ± 0.017
ARR	24	0.299 ± 0.011	0.294 ± 0.016	0.057 ± 0.006	0.058 ± 0.007	0.057 ± 0.007	0.059 ± 0.006	0.055 ± 0.006	0.061 ± 0.007	0.057 ± 0.007	0.079 ± 0.011	0.050 ± 0.006	0.067 ± 0.009	–	0.059 ± 0.008	0.074 ± 0.01	0.109 ± 0.017	0.107 ± 0.018
CSP	18	0.297 ± 0.009	0.309 ± 0.013	0.029 ± 0.004	0.029 ± 0.004	0.029 ± 0.005	0.032 ± 0.004	0.026 ± 0.004	0.031 ± 0.005	0.027 ± 0.004	0.054 ± 0.007	0.028 ± 0.004	0.058 ± 0.008	0.059 ± 0.008	–	0.036 ± 0.004	0.110 ± 0.015	0.107 ± 0.015
MON	19	0.298 ± 0.009	0.306 ± 0.011	0.008 ± 0.003	0.007 ± 0.002	0.008 ± 0.003	0.012 ± 0.002	0.006 ± 0.002	0.007 ± 0.002	0.005 ± 0.002	0.034 ± 0.007	0.015 ± 0.003	0.056 ± 0.009	0.074 ± 0.01	0.036 ± 0.004	–	0.116 ± 0.020	0.113 ± 0.020
TB_UK	19	0.306 ± 0.010	0.321 ± 0.014	0.099 ± 0.017	0.101 ± 0.018	0.095 ± 0.015	0.098 ± 0.017	0.097 ± 0.016	0.106 ± 0.018	0.101 ± 0.017	0.122 ± 0.017	0.064 ± 0.012	0.097 ± 0.016	0.109 ± 0.017	0.110 ± 0.015	0.116 ± 0.02	–	0.003 ± 0.008
TB_US	17	0.310 ± 0.012	0.323 ± 0.016	0.096 ± 0.017	0.098 ± 0.017	0.092 ± 0.016	0.095 ± 0.018	0.094 ± 0.016	0.104 ± 0.017	0.099 ± 0.017	0.12 ± 0.018	0.060 ± 0.011	0.094 ± 0.017	0.107 ± 0.018	0.107 ± 0.015	0.113 ± 0.02	0.003 ± 0.008	–

*N –* number of individual samples.

*H*_*E*chr_ – expected heterozygosity per chromosome, mean ± standard deviation.

*H*_Ochr_ – observed heterozygosity per chromosome, mean ± standard deviation.

*F*_*ST*_ – Wright's fixation index between populations.

Linkage disequilibrium (LD) analysis (Fig. 1, A) displayed higher values of *R*^*2*^ coefficient in population 39 comparing to other Zhabe populations. Foreign breeds except Mongolian horses have shown much higher values; the highest *R*^*2*^ coefficients and slower LD decay was in two Thoroughbred populations (Fig. 1, B). Mongolian horses had closer LD values to Zhabe horses than to other foreign breeds.

**Fig. 2:**
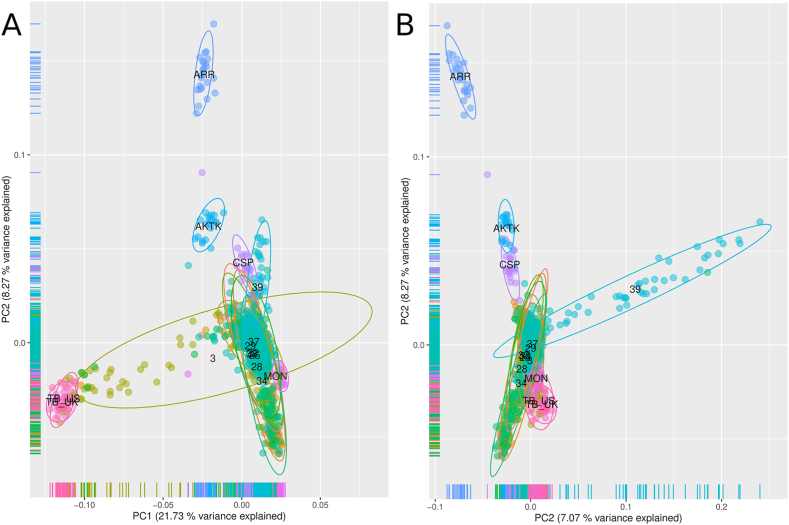
Principal component analysis of Zhabe horses in comparison to five foreign breeds; A) Scatterplot of components 1 and 2; B) Scatterplot of components 2 and 3. Population codes according Table 1.

Principal component analysis (Fig. 2) have shown a presence of outlying genotypes of Zhabe (populations 3 and 33) shifted towards Thoroughbred whereas most Zhabe samples have been combined into a single dense cluster. Principal component 1 (PC1) explaining 21.73 % of total variance contributes the most to discrimination between Zhabe horses and Thoroughbred, and PC2 with 8.27 % of explained variance separated Arabian horses. Whereas first two components place population 39 close to other Zhabe horses, PC3 has shown its significant deviation. Mongolian horses were placed close to Zhabe. Akhal-Teke and Caspian horses formed distinct cluster close to Zhabe.

**Fig. 3:**
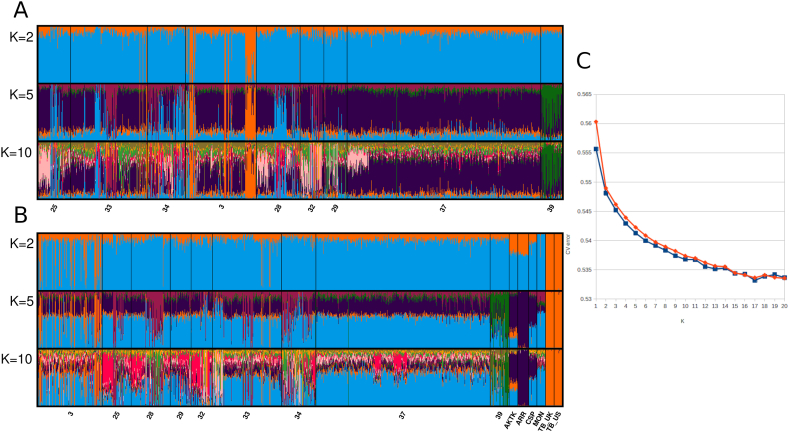
Results of ADMIXTURE analysis of Zhabe horses in comparison to five foreign breeds; A) Data on Zhabe horses, 43,422 SNP; B) Data on Zhabe horses combined with five foreign breeds (24,764 SNP); C) Plot of cross-validation error for data A (blue line) and B (red line). Population codes according Table 1.

ADMIXTURE analysis was performed for numbers of expected clusters *K* from 1 to 20. Cross-validation test have not revealed the best *K* value; although the error value was the lowest at *K* = 17, the absence of following inclining trend on the plot makes its selection doubtful (Fig. 3, C). Fig. 3A and B, demonstrates patterns for *K=*2, 5, and 10 selected based on examination of diagrams as demonstrating most significant features of the obtained results. With *K=*2 outlying genotypes persistent across all *K* configurations have been identified (shown orange in all diagrams). Comparative analysis with foreign breed allowed to attribute these individuals to inclusion of Thoroughbred into selection process. Population 39 was classified as a unique cluster dissimilar to either Zhabe or foreign breeds. Higher *K* patterns reveal within population heterogeneity of Zhabe breeds.

**Fig. 4:**
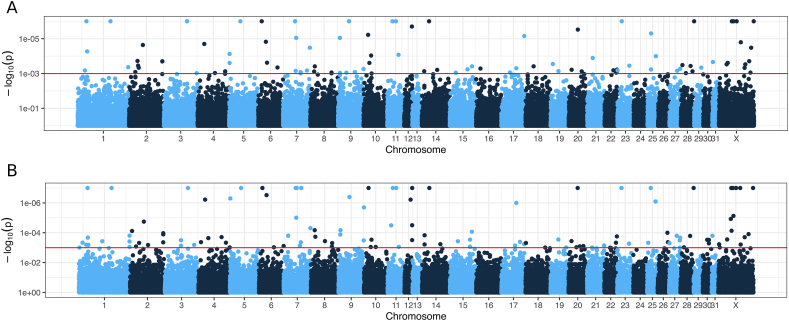
Manhattan plots of P-values adjusted by the Monte-Carlo permutation test from genome-wide association analysis for body size (A) and weight (B) in Zhabe horses. Red line indicates significance threshold *p=*0.001.

For genome wide association study, horse individuals identified as outlying genotypes by PCA and ADMIXTURE analysis were excluded from consideration. Total 823 animals were used for analysis, including 391 newly obtained genotypes and 432 genotypes from previously published data [37]. The summary of phenotypic data used is shown in Table 3. As a result, 126 SNPs had *p-*value adjusted by Monte-Carlo permutation test for association with body weight, and 99 SNPs for body size. All variants satisfying the selected significance threshold *P* < 0.001 were distributed occasionally, and no regions with high occurrence of significant SNPs were observed (Fig. 4). After variant annotation and filtering of the results, total 57 SNPs have been retained, including 14 and 23 SNPs associated only with body size and weight, correspondingly, and 20 variants associated with both traits (Table 4). Gene *CFI* (complement factor I) contained three revealed SNPs; for genes *DLG2, KIT*, and *PRKG2* two variants have been identified; other genes contained single SNPs. Most annotated genes were have been involved into various regulatory processes and signal transmission.

**Table 3:** Summary of phenotypic traits of Zhabe horse populations.

Population code	Sex (percentage of mares in the sample)	Age		Weight		HW		OBL		CC		CBC	
		mean	sd	mean	sd	mean	sd	mean	sd	mean	sd	mean	sd
25	92.19	8.111	3.399	417.328	41.505	142.422	2.308	146.719	4.022	173.406	7.272	18.805	0.568
28	91.86	6.209	3.505	394.128	38.763	141.372	3.002	145.977	3.700	173.012	6.934	17.878	0.659
29	93.48	5.435	2.177	359.326	61.695	139.348	4.729	142.391	7.120	168.891	8.817	17.413	0.896
3	91.43	6.993	3.771	397.679	52.221	141.157	3.740	146.036	5.756	171.571	8.259	18.050	0.896
32	100.00	n.d.	n.d.	n.d.	n.d.	n.d.	n.d.	n.d.	n.d.	n.d.	n.d.	n.d.	n.d.
33	100.00	7.993	2.272	388.901	12.641	139.605	1.479	143.645	1.673	164.757	2.258	17.688	0.372
34	89.33	8.507	0.891	401.320	28.991	140.293	2.198	144.427	2.579	167.160	5.514	18.600	0.539
37	96.88	3.786	1.657	396.997	44.904	141.882	2.838	145.743	3.456	169.322	5.589	17.732	0.870
39	80.95	4.190	1.194	380.286	37.050	140.119	2.596	143.810	3.763	168.976	4.176	17.788	0.678

nd. - no data available.

**Table 4:** Single nucleotide polymorhisms associated with horse weight and size annotated using VEP and DAVID tools.

Variant ID	Location	Gene	*P*-value		GO term – biological process
			Size	Weight	
BIEC2_316765	15:68252917	ALK	–	0.0008545	GO:0006468∼protein phosphorylation
AX-103265166	2:55284182	BNIP3L	2.3 × 10^−5^	1.80 × 10^−5^	GO:0016239∼positive regulation of macroautophagy
AX-104903715	4:93924679	BRAF	0.0007523	0.0004619	GO:0000165∼MAPK cascade
BIEC2_946110	6:29507974	CACNA1C	0.0002397	–	GO:0002520∼immune system development
BIEC2_140284	11:13962473	CACNG4	–	3.181 × 10^−5^	GO:0019226∼transmission of nerve impulse
Affx-102683437	4:79003820	CADPS2	–	0.0001952	GO:0006887∼exocytosis
AX-104609861	6:85773796	CCT2	–	0.0007669	GO:0006457∼protein folding
BIEC2_508762	2:116459352	CFI	–	0.0004362	GO:0006508∼proteolysis
BIEC2_508766	2:116465026	CFI	0.0002012	0.0001068	GO:0006508∼proteolysis
BIEC2_508769	2:116466442	CFI	0.0001972	0.0001277	GO:0006508∼proteolysis
TBIEC2_331745	15:68417648	CLIP4	–	0.0002872	GO:0031122∼cytoplasmic microtubule organization
Affx-101130275	4:60087882	CPVL	0.0009387	–	GO:0006508∼proteolysis
BIEC2_700308	27:2259858	CSGALNACT1	–	0.0004961	GO:0001958∼endochondral ossification
BIEC2_725599	28:5564261	CSRP2	0.0003285	–	GO:0045214∼sarcomere organization
AX-103706694	1:120490380	CYP11A1	1 × 10^−6^	1.00 × 10^−7^	GO:0006700∼C21-steroid hormone biosynthetic process
TBIEC2_498975	2:40275747	DISP3	0.0004661	–	GO:0045665∼negative regulation of neuron differentiation
AX-103721396	7:63690864	DLG2	0.0008281	1.00 × 10^−7^	GO:0007268∼chemical synaptic transmission
BIEC2_1004624	7:62981215	DLG2	0.0009895	–	GO:0007268∼chemical synaptic transmission
BIEC2_722382	27:39199656	DLGAP2	–	0.0002175	GO:0023052∼signaling
AX-103583267	26:37333692	DSCAM	–	0.0004288	GO:0007156∼homophilic cell adhesion via plasma membrane adhesion molecules
Affx-103045508	2:27182213	EPB41	0.0008206	–	GO:0007049∼cell cycle
TBIEC2_889720	4:8502727	EPDR1	–	0.0004253	GO:0007160∼cell-matrix adhesion
BIEC2_852365	3:115596394	EVC	0.0009599	–	GO:0003416∼endochondral bone growth
BIEC2_416905	18:63653165	FAM171B	–	0.0009746	GO:0001525∼angiogenesis
Affx-102845142	28:31765971	FBXO7	0.0003695	0.0001627	GO:0000422∼mitophagy
BIEC2-472336	2:37787520	FHAD1	0.0003518	−0.0004809	–
BIEC2_14481	1:32086433	FRAT1	5.366 × 10^−5^	0.0006401	GO:0090263∼positive regulation of canonical Wnt signaling pathway
Affx-102013817	17:62060245	GPC5	–	0.0004061	GO:0016477∼cell migration
Affx-102676537	6:24907627	HDAC4	1.5 × 10^−5^	3.00 × 10-^7^	GO:0000122∼negative regulation of transcription from RNA polymerase II promoter
BIEC2_14318	1:30611785	HPSE2	–	0.0002113	GO:0008283∼cell proliferation
ITGA2B_19245752_GT	11:19245752	ITGA2B	1 × 10^−6^	–	GO:0001525∼angiogenesis
KIT_79540741_W16	3:79540741	KIT	1 × 10^−6^	1.00 × 10^−7^	GO:0001541∼ovarian follicle development
KIT_79580000_W7	3:79580000	KIT	1 × 10^−6^	1.00 × 10^−7^	GO:0001541∼ovarian follicle development
BIEC2_320442	15:75799411	LDAH	0.0003885	–	GO:0019915∼lipid storage
Affx-101492057	10:19641430	NOSIP	6 × 10^−6^	1.00 × 10^−7^	–
AX-104335949	5:40161837	NUP210L	1 × 10^−6^	1.00 × 10^−7^	–
PAX3_11199140_SW4	6:11199140	PAX3	1 × 10^−6^	1.00 × 10^−7^	GO:0006355∼regulation of transcription
AX-104963491	12:30951642	PITPNM1	2 × 10^−6^	1 × 10^−7^	GO:0015914∼phospholipid transport
BIEC2_887173	5:2222786	PM20D1	0.0002478	–	GO:0006520∼cellular amino acid metabolic process
BIEC2_783022	3:57169047	PRKG2	–	0.0007432	GO:0006468∼protein phosphorylation
BIEC2_783026	3:57171222	PRKG2	–	0.0003162	GO:0006468∼protein phosphorylation
BIEC2_885537	4:107978404	PTPRN2	–	0.0009618	GO:0006470∼protein dephosphorylation
BIEC2_591905	22:33100482	PTPRT	0.0006552	–	GO:0006470∼protein dephosphorylation
Affx-103079316	12:30867790	RAD9A	–	3.127 × 10^−5^	GO:0000076∼DNA replication checkpoint
AX-104886193	25:31276468	RALGPS1	0.0001003	8.00 × 10^−7^	GO:0007264∼small GTPase mediated signal transduction
Affx-102836572	17:50354202	SLAIN1	0.0004986	1.00 × 10^−6^	GO:0007020∼microtubule nucleation
SLC36A1_25884457_Champagne	14:25884457	SLC36A1	1 × 10^−6^	1.00 × 10^−7^	GO:0015808∼L-alanine transport
BIEC2_11418	1:24106986	SORCS1	0.0006665	–	GO:0006892∼post-Golgi vesicle-mediated transport
BIEC2_954544	6:50669754	SOX5	–	0.0009407	GO:0006357∼regulation of transcription from RNA polymerase II promoter
BIEC2_1011868	7:87211576	SOX6	0.0006732	–	GO:0006357∼regulation of transcription from RNA polymerase II promoter
AX-104226721	9:36874369	SPIDR	1 × 10p	4.00 × 10^−7^	GO:0000724∼double-strand break repair via homologous recombination
AX-104855978	28:39009737	TEF	0.0007493	–	GO:0006357∼regulation of transcription from RNA polymerase II promoter
BIEC2_486399	2:69215803	TLL1	–	0.0006598	GO:0006508∼proteolysis
BIEC2_1004550	7:62165511	TMEM126B	–	0.0004348	GO:0032094∼response to food
BIEC2_195234	12:24939247	TMEM138	–	6 × 10^−7^	–
AX-103728731	9:84365361	ZC3H3	0.0005481	2.00 × 10^−6^	–
Affx-101705964	10:25531136	ZNF787	0.0002468	–	GO:0000122∼negative regulation of transcription from RNA polymerase II promoter

## 4 Discussion

The obtained results on genetic structure of Zhabe horses are in line with our previous findings [37]. As we discussed in the mentioned paper, the specific conditions of traditional nomadic husbandry could affect genetic composition of Kazakh horses. Almost free grazing of horses on summer and winter pastures with low control of mating by the herders have been closer to the ways of existence of horses in the wild nature rather than horse breeds under strictly controllable stable conditions. Here we have examined 443 newly genotyped horse individuals in addition to the previously analyzed and reported horse samples (616) to have a deeper look on genetic structure of Zhabe horses in comparison with several foreign horse breeds, Akhal-Teke, Arabian, Caspian, Mongolian, and Thoroughbred (data by Ref. [41]). These particular breeds have been selected under specific considerations: Mongolian and Caspian horses have previously shown high genetic similarity to Kazakh horses [37]; Akhal-Teke breed was selected as another ancient breed with Central Asian origin; Arabian and Thoroughbred horses have been selected as reference well known breeds of stable maintenance and high breeding control; moreover, Thoroughbred horses are known to be involved to later breeding practice for improvement of Kazakh horses. Indeed, the presence of presumably hybrid genotypes between Zhabe and Thoroughbred horses was revealed by PCA and ADMIXTURE analysis. Population 3 (Kalka) which included the most of Thoroughbred hybrids was also shown closer to this breed by *F*_*ST*_ analysis. In comparison, Zhabe horses display lower degree of LD comparing to foreign breeds. Such population genetics concepts as LD decay and ROH are considered as selection signatures in domestic animals [47]. Comparing to foreign breeds, we have shown in our study that Zhabe horses indeed have these signatures less expressed. Lower LD parameters indicate higher individual variability within populations which is also illustrated by results of ADMIXTURE analysis. As Fig. 3 shows, besides individuals with identified admixture with Thoroughbred horses, higher *K* patterns demonstrate additional within populaion variability which is, however, is not associated with particluar populations. It was shown previously that such internal diversity within populations may be caused by the influence of groups of closely related horses, e.g. originated from the same productive sires [48]. However, the lack of pedigree data on Kazakh horses due to low control of mating makes it impossible to evaluate such possible substructure in this study.

Population 39 representing unique white horses ‘Zhetysu Asyly’ posed a special interest. This lineage is a result of amateur selection and have not been an object of scientific evaluation to date. Although it was stated by the breeder (personal communication) that this breed have been established based on Zhabe and other Kazakh horses, no documents on the selection history are available. Our results reveal surprisingly high level of differentiation of population 39 comparing to Zhabe horses. Patterns of LD and *F*_*ST*_ of these horses is more similar to foreign breeds, however PCA, *F*_*ST*_ and ADMIXTURE analysis place this population as unique cluster. Considering strict artificial selection towards color, the observed differences should be considered selective signatures. If the declared assumption about their origin from Kazakh horses is correct, their genetic composition could be an example of rapid transformation of animal's genome under pressure of targeted selection. However, lack of reliable descriptive and genealogical data makes possible assumption speculative. Thus, so called ‘Zhetysy Asyly’ horses are a potential object for further deeper investigation.

Previously, we have attempted GWAS to identify variants associated with body weight and size in Kazakh horses [37]. The results of that work have demonstrated the absence of genomic regions strongly associated with the considered body traits. The variants with significant associations were distributed occasionally and did not form notable islands corresponding to genomic regions to increased association. Moreover, no SNPs associated with known major horse body size factors, genes *LCORL*, *NCARG*, and *ZFAT* [31,32,34], have been revealed. In the present study, we obtained similar results for selection of Zhabe horses. Interestingly, the loci identified here showed no correspondence to previously reported ones. Such a distribution of occasional SNPs could be a sign of the absence of strong selection signatures for body size and weight in Kazakh horses and, particularly, Zhabe horse type. Thus, these traits of Zhabe horses, as well as other Kazakh horse varieties, could be determined by environment to higher extent than genetic factors.

Along with the previous findings, our results illustrate specific genetic properties of Kazakh horses. Traditional nomadic ways of horse breeding have lead to formation of unique landrace with low presence of selection signatures. We could suggest that this lack of dominating genetic factors developed by selection is the reason of high versatility and adaption of Kazakh horses to a range of conditions of Central Asia, from steppes to mountains. On the other hand, the genetic identity of Kazakh horses is vulnerable before targeted breeding practices and hybridization. As we have shown here (Figs. 2 and 3), relatively recent hybridization events involving Thoroughbred horses have left distinctive signature allowing to identify source of admixture. Horses “Zhetysu Asyly” (population 39) resulted from targeted selection of Kazakh horses for white color demonstrate significant changes in their genetic composition from Zhabe horses and, considering low variability between breeds [37], Kazakh horses in general. Thus, Kazakh horses and, particularly, Zhabe type should be considered a unique legacy landrace requiring special protection. The obtained results provide genetic data on the Kazakh horses of Zhabe type which are traditional but yet understudied horse breed. To conserve their genetic composition and properties the breeding programs should be focused on the internal genetic resources of Kazakh horses rather than hybridization with distant breeds. Still, Kazakh horses may be a valuable genetic resource for developing new horse breeds due to their unique properties and adaption to environmental conditions.

## 5 Conclusion

The present work provides important insights into the genetic structure of Zhabe horses and other Kazakh horse populations. The analysis revealed that traditional nomadic breeding practices, characterized by minimal human interference, have shaped these horses into a unique landrace with low selection signatures and high genetic diversity. Population 39, the white horses known as ‘Zhetysu Asyly,’ displayed significant genetic differentiation, likely due to targeted breeding for coat color. The findings highlight the importance of conserving the genetic integrity of Kazakh horses, particularly Zhabe, to preserve their adaptability and historical significance, while recognizing their potential as a genetic resource for future breeding programs.

## CRediT authorship contribution statement

**Alexandr Pozharskiy:** Writing – original draft, Visualization, Formal analysis, Data curation. **Indira Beishova:** Supervision, Resources, Project administration, Conceptualization. **Askar Nametov:** Writing – review & editing, Supervision. **Alzhan Shamshidin:** Resources. **Tatyana Ulyanova:** Methodology, Investigation. **Alexandr Kovalchuk:** Methodology, Investigation. **Vadim Ulyanov:** Investigation. **Malika Shamekova:** Resources, Investigation. **Gulmira Bekova:** Methodology, Investigation. **Dilyara Gritsenko:** Writing – review & editing, Supervision, Project administration, Conceptualization.

## Ethics statement

The study was conducted according to the guidelines of the European Convention for the Protection of Vertebrate Animals used for Experimental and Other Scientific Purposes, and approved by the Local Ethics Committee of Zhengir Khan West-Kazakhstan Agrarian Technical University (protocol № 1, April 4, 2022).

## Data availability statement

The raw data supporting the conclusions of this article will be made available by the authors on request.

## Funding

This research was funded by the Ministry of Science and Higher Eduction of the Republic of Kazakhstan within the framework of the of the research project AP14870614 «Genetic marking of productive traits of the Kazakh horse of the Dzhabe type based on genome-wide coverage SNP genotyping»

## Declaration of competing interest

The authors declare that they have no known competing financial interests or personal relationships that could have appeared to influence the work reported in this paper.

## References

[1] Frachetti M., Benecke N. (2009). From sheep to (some) horses: 4500 years of herd structure at the pastoralist settlement of Begash (south-eastern Kazakhstan). Antiquity.

[2] Outram A.K., Stear N.A., Bendrey R., Olsen S., Kasparov A., Zaibert V., Thorpe N., Evershed R.P. (2009). The earliest horse harnessing and milking. Science.

[3] Outram A., Bendrey R., Evershed R.P., Orlando L., Zaibert V.F. (2021). Rebuttal of taylor and barrón-ortiz 2021 rethinking the evidence for early horse domestication at botai.

[4] Nurushev M.Zh, Zaybert V.F. (2018). Steppes of Northern Eurasia:Proceedings of the 8th International Symposium.

[5] Barmintsev YuN. (1958). Experience of Zootechnical Study of the Poroblem of Breed formation[Evoljucija Konskih Porod V Kazahstane. Opyt Zootehnicheskogo Issledovanija Problemy Porodoobrazovanija].

[6] Dmitriev N.G., Ėrnst L.K. (1989). Animal Genetic Resources of the USSR.

[7] Dyussegaliyev M.Zh (2022). The genotypes of herd horses of the west region of Kazakhstan. The Agriculture and Ecosystems in Modern World: Regional and Inter countries’ research.

[8] Nechayev I.N., Zhumagul A.E., Sizonov G.V., Zhaytapov T., Kikebayev N.A., Nurushev M.Zh (2005).

[9] Kargayeva M.T., Baimukanov D.A., Dzhunisov A.M., Alikhanov О. (2019). On meat productivity of young Kazakh horses of the Jabe type on the Mangyshlak peninsula. Bulletin of KhSU named after. N.F. Katanova.

[10] Chang C. (2015). The Ecology of Pastoralism.

[11] Mohammadi A., Nassiry M.R., Mosafer J., Mohammadabadi M.R., Sulimova G.E. (2009). Distribution of BoLA-DRB3 allelic frequencies and identification of a new allele in the Iranian cattle breed Sistani (Bos indicus). Russ. J. Genet..

[12] Bordbar F., Mohammadabadi M., Jensen J., Xu L., Li J., Zhang L. (2022). Identification of candidate genes regulating carcass depth and hind leg circumference in simmental beef cattle using Illumina bovine beadchip and next-generation sequencing analyses. Animals.

[13] Mohammadabadi M. (2016). Inter-simple sequence repeat loci associations with predicted breeding values of body weight in kermani sheep. Genetics in the 3rd Millennium.

[14] Javanmard A., Mohammadabadi M.R., Zarrigabayi G.E., Gharahedaghi A.A., Nassiry M.R., Javadmansh A., Asadzadeh N. (2008). Polymorphism within the intron region of the bovine leptin gene in Iranian Sarabi cattle (Iranian Bos taurus). Russ. J. Genet..

[15] Alinaghizadeh R., Mohammed Abadi M., Moradnasab B.S. (2007). Kappa-casein gene study in Iranian sistani cattle breed (Bos indicus) using PCR-RFLP. Pakistan J. Biol. Sci..

[16] Mousavizadeh A., Abadi M.M., Torabi A., Nassiry R., Ghiasi H., Koshkoieh A.E. (2009). Genetic polymorphism at the growth hormone locus in Iranian Talli goats by polymerase chain reaction-single strand conformation polymorphism (PCR-SSCP). Iran. J. Biotechnol..

[17] Nejad F.M., Mohammadabadi M., Roudbari Z., Gorji A.E., Sadkowski T. (2024). Network visualization of genes involved in skeletal muscle myogenesis in livestock animals. BMC Genom..

[18] Mohammadabadi M.R., Soflaei M., Mostafavi H., Honarmand M. (2011). Using PCR for early diagnosis of bovine leukemia virus infection in some native cattle. Genet. Mol. Res..

[19] Molaei Moghbeli S., Barazandeh A., Vatankhah M., Mohammadabadi M. (2013). Genetics and non-genetics parameters of body weight for post-weaning traits in Raini Cashmere goats. Trop. Anim. Health Prod..

[20] Mohammadabadi M.R., Tohidinejad F. (2017). Characteristics determination of rheb gene and protein in raini cashmere goat. Iran. J. Appl. Anim. Sci..

[21] Norouzy A., Nassiry M.R., Eftekhari Shahrody F., Javadmanesh A., Mohammad Abadi M.R., Sulimova G.E. (2005). Identification of bovine leucocyte adhesion deficiency (BLAD) carriers in holstein and Brown Swiss AI bulls in Iran. Russ. J. Genet..

[22] Sulimova G.E., Azari M.A., Rostamzadeh J., Mohammad Abadi M.R., Lazebny O.E. (2007). κ-casein gene (CSN3) allelic polymorphism in Russian cattle breeds and its information value as a genetic marker. Russ. J. Genet..

[23] Molina A., Valera M., Dos Santos R., Rodero A. (1999). Genetic parameters of morphofunctional traits in Andalusian horse. Livest. Prod. Sci..

[24] Suontama M., Saastamoinen M.T., Ojala M. (2009). Estimates of non-genetic effects and genetic parameters for body measures and subjectively scored traits in Finnhorse trotters. Livest. Sci..

[25] Tamioso P.R., Cosmo T.R., Pimentel C.M.M., Dias L.T., Teixeira R. de A. (2012). Heritability estimates for body weight and height at withers in Brazilian army horses. Ciência Rural..

[26] Akimbekov A.R., Baimukanov D.A. (2017). Breeding of Seletinian stud farm type of Kazakh dzhabe Horses. Izvestiya TSHA.

[27] Baimukanov D.A., Akimbekov A.R., Aubakirov KhA., Kenzhekhodzhayev M.D., Alikhanov О., Nurmakhanbetov D. (2017).

[28] Kalbfleisch T.S., Rice E.S., DePriest M.S., Walenz B.P., Hestand M.S., Vermeesch J.R., O′Connell B.L., Fiddes I.T., Vershinina A.O., Saremi N.F., Petersen J.L., Finno C.J., Bellone R.R., McCue M.E., Brooks S.A., Bailey E., Orlando L., Green R.E., Miller D.C., Antczak D.F., MacLeod J.N. (2018). Improved reference genome for the domestic horse increases assembly contiguity and composition. Commun. Biol..

[29] McCue M.E., Bannasch D.L., Petersen J.L., Gurr J., Bailey E., Binns M.M., Distl O., Guérin G., Hasegawa T., Hill E.W., Leeb T., Lindgren G., Penedo M.C.T., Røed K.H., Ryder O.A., Swinburne J.E., Tozaki T., Valberg S.J., Vaudin M., Lindblad-Toh K., Wade C.M., Mickelson J.R. (2012). A high density SNP array for the domestic horse and extant Perissodactyla: utility for association mapping, genetic diversity, and phylogeny studies. PLoS Genet..

[30] Petersen J.L., Mickelson J.R., Rendahl A.K., Valberg S.J., Andersson L.S. (2013). Genome-wide analysis reveals selection for important traits in domestic horse breeds. PLoS Genet..

[31] Signer-Hasler H., Flury C., Haase B., Burger D., Simianer H., Leeb T., Rieder S. (2012). A genome-wide association study reveals loci influencing height and other conformation traits in horses. PLoS One.

[32] Makvandi-Nejad S., Hoffman G.E., Allen J.J., Chu E., Gu E., Chandler A.M., Loredo A.I., Bellone R.R., Mezey J.G., Brooks S.A., Sutter N.B. (2012). Four loci explain 83% of size variation in the horse. PLoS One.

[33] Tetens J., Widmann P., Kühn C., Thaller G. (2013). A genome-wide association study indicates LCORL/NCAPG as a candidate locus for withers height in German Warmblood horses. Anim. Genet..

[34] Tozaki T., Kikuchi M., Kakoi H., Hirota K., Nagata S. (2017). A genome-wide association study for body weight in Japanese Thoroughbred racehorses clarifies candidate regions on chromosomes 3, 9, 15, and 18. JES (J. Environ. Sci.).

[35] Kargayeva M.T., Baimukanov D.A., Nurbaev S.D., Baimukanov A.D., Alikhanov О., Yusupbayev Zh (2020). Identification of Kazakh horses by microsatelite DNA using modern analytical methods. Bulletin of NAS RK.

[36] Seleuova L.A., Naimanov D.K., Jaworski Z., Aubakirov M.Zh, Mustafin M.K., Mustafin B.M., Safronova O.S., Baktybayev G.T., Turabayev A.T., Domatski V.N. (2018). Population genetic characteristic of horses of Mugalzhar breed by STR-markers. Biomed. Res..

[37] Pozharskiy A., Abdrakhmanova A., Beishova I., Shamshidin A., Nametov A., Ulyanova T., Bekova G., Kikebayev N., Kovalchuk A., Ulyanov V., Turabayev A., Khusnitdinova M., Zhambakin K., Sapakhova Z., Shamekova M., Gritsenko D. (2023). Genetic structure and genome-wide association study of the traditional Kazakh horses. Animal.

[38] Purcell S., Neale B., Todd-Brown K., Thomas L., Ferreira M.A.R., Bender D., Maller J., Sklar P., De Bakker P.I.W., Daly M.J., Sham P.C. (2007). PLINK: a tool set for whole-genome association and population-based linkage analyses. Am. J. Hum. Genet..

[39] Alexander D.H., Lange K. (2011). Enhancements to the ADMIXTURE algorithm for individual ancestry estimation. BMC Bioinf..

[40] Kopelman N.M., Mayzel J., Jakobsson M., Rosenberg N.A., Mayrose I. (2015). Clumpak: a program for identifying clustering modes and packaging population structure inferences across K. Molecular Ecology Resources.

[41] Petersen J.L., Mickelson J.R., Cothran E.G., Andersson L.S., Axelsson J., Bailey E., Bannasch D., Binns M.M., Borges A.S., Brama P., Machado A. da C., Distl O., Felicetti M., Fox-Clipsham L., Graves K.T., Guérin G., Haase B., Hasegawa T., Hemmann K., Hill E.W., Leeb T., Lindgren G., Lohi H., Lopes M.S., McGivney B.A., Mikko S., Orr N., Penedo M.C.T., Piercy R.J., Raekallio M., Rieder S., Røed K.H., Silvestrelli M., Swinburne J., Tozaki T., Vaudin M., Wade C.M., McCue M.E. (2013). Genetic diversity in the modern horse illustrated from genome-wide SNP data. PLoS One.

[42] R Core Team (2019). R: a language and environment for statistical computing. https://www.r-project.org/.

[43] Potts A., White T.W., Stachniss C., Kendall M., Beaulieu J., Meara B.O. (2021).

[44] McLaren W., Gil L., Hunt S.E., Riat H.S., Ritchie G.R.S., Thormann A., Flicek P., Cunningham F. (2016). The ensembl variant effect predictor. Genome Biol..

[45] Huang D.W., Sherman B.T., Lempicki R.A. (2009). Systematic and integrative analysis of large gene lists using DAVID bioinformatics resources. Nat. Protoc..

[46] Sherman B.T., Hao M., Qiu J., Jiao X., Baseler M.W., Lane H.C., Imamichi T., Chang W. (2022). DAVID: a web server for functional enrichment analysis and functional annotation of gene lists (2021 update). Nucleic Acids Res..

[47] Saravanan K.A., Panigrahi M., Kumar H., Bhushan B., Dutt T., Mishra B.P. (2020). Selection signatures in livestock genome: a review of concepts, approaches and applications. Livest. Sci..

[48] Gmel A.I., Mikko S., Ricard A., Velie B.D., Gerber V., Hamilton N.A., Neuditschko M. (2024). Using high-density SNP data to unravel the origin of the Franches-Montagnes horse breed. Genet. Sel. Evol..

